# Dutch family physicians’ awareness of cognitive impairment among the elderly

**DOI:** 10.1186/s12877-015-0105-1

**Published:** 2015-08-27

**Authors:** Pim van den Dungen, Eric P. Moll van Charante, Peter M. van de Ven, Gerbrand Foppes, Jos P. C. M. van Campen, Harm W. J. van Marwijk, Henriëtte E. van der Horst, Hein P. J. van Hout

**Affiliations:** Department of General Practice and Elderly Care Medicine, EMGO Institute for Health and Care Research, VU University Medical Center Amsterdam, Van der Boechorststraat 7, 1081 BT Amsterdam, The Netherlands; Department of General Practice, Academic Medical Center, University of Amsterdam, Amsterdam, The Netherlands; Department of Epidemiology and Biostatistics, VU University Medical Center Amsterdam, Amsterdam, The Netherlands; Department of Geriatric Medicine, Slotervaart Hospital, Amsterdam, The Netherlands; Primary Care Research Centre, Institute of Population Health, University of Manchester, Manchester, UK

## Abstract

**Background:**

Dementia is often not formally diagnosed in primary care. To what extent this is due to family physicians’ (FPs) watchful waiting, reluctance to diagnose or to their unawareness of the presence of cognitive impairment is unclear. The objective of this study was to assess FPs’ awareness of cognitive impairment by comparing their evaluation of the absence or presence of cognitive impairment in older patients without an established diagnosis of dementia, with a reference test of cognitive functioning. In addition, we assessed which patient characteristics were associated with con- and discordance between FPs’ evaluation of cognition and results of the reference test.

**Methods:**

The design was a nested diagnostic study. FPs (*n* = 29) of 15 primary care practices classified the cognitive status of all their patients ≥ 65 years of age (*n* = 7865) into four categories, based on recollection and medical records. All patients categorized as ‘possible cognitive impairment or dementia’ and a sample of patients categorized as ‘no signs of cognitive impairment’ randomly selected to match age and gender were offered to receive a reference test of cognitive function (the CAMCOG) to verify the FPs’ label. This reference test could yield three outcomes: no cognitive impairment, amnestic mild cognitive impairment (aMCI) or dementia. Reference test results were weighted back to the original samples to provide estimates for the correct categorization of elderly as ‘possible cognitive impairment or dementia’ (positive predictive value [PPV]) and ‘no signs of cognitive impairment’ (negative predictive value [NPV]). Cognitive functioning was not assessed for patients evaluated by FPs as ‘probable dementia’ and ‘unknown or no recent contact’. Characteristics associated with the con- or discordance of the FPs’ classification and the reference test were assessed using logistic regression.

**Results:**

Complete reference test results were obtained from 318 elderly. FPs labeled 8.3 % of elderly ‘possible cognitive impairment or dementia’. The PPV of this label for a CAMCOG score suggestive of dementia *or* aMCI was 47.1 % (95 %-confidence interval: 43.5 – 62.4 %). FPs labeled 83.7 % ‘no signs of cognitive impairment’. The 1-NPV of this label for a CAMCOG score suggestive of dementia *or* aMCI was 12.5 % (95 %-CI 8.2 – 16.8 %). FPs labeled 3.6 % as ‘probable dementia’ and 4.5 % as ‘unknown or no recent contact’. The odds that FPs’ suspicion of cognitive impairment were confirmed by the CAMCOG were higher if persons were ADL dependent (OR 2.24 [95 %-CI 1.16 – 4.35]). The odds of FPs being unaware of the presence of cognitive impairment were higher in the older elderly (OR 1.15 [95 %-CI 1.09 – 1.23] per year).

**Conclusion:**

Evaluation of FPs’ classification of the global cognitive function of elderly without a firm diagnosis of dementia showed both over- and unawareness of the presence of cognitive impairment. FPs were more often unaware of cognitive impairment in the older elderly.

**Electronic supplementary material:**

The online version of this article (doi:10.1186/s12877-015-0105-1) contains supplementary material, which is available to authorized users.

## Background

Late or missed dementia diagnoses are not uncommon in the primary care setting. The percentage of all dementia cases within a practice that are diagnosed ranges from 14 to 33 % for mild, and from 38 to 71 % for moderate to severe dementia [1]. Currently, there is a lot of emphasis on early diagnosis of dementia, mostly by policy makers, and FPs are sometimes ‘accused’ of diagnostic reluctance or said to be in need of more training [2–5]. This plea for ‘more dementia diagnoses’ is countered by people stating that the diagnosis of dementia is a cumulative process and that FPs weigh carefully whether and when this high impact diagnosis is opportune in every individual case [3].

What is missing in this discussion about whether or not FPs should proceed more rigorously, is information about how often FPs are at all *aware* of cognitive impairment [6, 7]. Another thing missing is information about the predictive value of such awareness for the actual presence or absence of cognitive impairment. Earlier studies on FPs’ diagnostic accuracy provide only limited information since their outcomes (e.g. sensitivity) always concerned already diagnosed patients, whereas diagnostic gain can only be achieved in individuals without an established diagnosis of dementia [1]. Possible outcomes of diagnostic evaluation of cognition by FPs comprise dementia, mild cognitive impairment, cognitive functioning normal for age or other causes for memory impairment such as affective disorders [8].

The aim of the current study was to estimate FPs’ awareness of the presence or absence of cognitive impairment and dementia among older persons without an established diagnosis of dementia by comparing their evaluation of the cognitive function of their older patients with a reference test. In addition, we assessed which patient characteristics were associated with con- and discordance between FPs’ evaluation of cognition and reference test results.

## Methods

### Design

We used a prospective nested design to study the diagnostic accuracy of FPs’ classification of the cognitive status of the older persons in their practices. Biesheuvel et al. described this design in detail [9, 10].

This study was a sub-study within a cluster RCT [11].

### Cognitive classification by FPs

At baseline the FPs of the 15 Primary Care Practices (PCPs) were provided with a list of all their patients aged 65 and above and asked to classify them in one of the following four categories:

- ‘no signs of cognitive impairment’ (negative index test)
- ‘possible cognitive impairment or dementia’ (positive index test)
- ‘probable dementia’
- ‘unknown or no recent contact’

FPs categorized based on their recollection. They were allowed to check their electronic medical records (EMR) if they wanted, but not to perform additional cognitive tests. There was no feedback to patients about the categorization.

### The index test

Since we were particularly interested in FPs’ awareness of cognitive impairment in cases where the diagnosis was not yet evident, we aimed to validate the label ‘possible cognitive impairment or dementia’ (positive index test). To explore unawareness, we validated the label ‘no signs of cognitive impairment’ (negative index test). Since previous literature demonstrated a high validity of the label ‘probable dementia’ by FPs, we assumed this would also apply to this classification by the FPs who participated in this study and chose not to validate this label [1].

### Determinants of cognitive classification by the FPs

A post hoc analysis was performed to assess whether the following patient characteristics were associated with being classified as ‘possible cognitive impairment or dementia’ or ‘no signs of cognitive impairment’: mental health status and quality of life (MH5, EQ-5D, QoL-AD), number of comorbidities, the presence of chronic diseases, the presence of psychiatric disorders, whether living alone or together and level of ADL and instrumental-ADL dependency (Katz).

### The reference standard

We needed a reference test that differentiated between normal cognition for age and cognitive impairment, including aMCI and dementia. We chose the Cambridge Cognitive Examination (CAMCOG) as a reference standard as it meets this criterion and has good reliability and psychometric properties [12, 13]. The sensitivity and specificity of this instrument for dementia as assessed by clinically trained experts using the DSM IV criteria are 93 and 87 %, respectively; and for amnestic MCI (aMCI) 78 and 74 %, respectively [12–15]. The CAMCOG was administered by trained interviewers blinded to the FPs’ classification (*n* = 5), and scored according to the CAMCOG scoring guideline. We used established age and education specific cut-offs for optimal test accuracy. The cut-off for dementia for individuals younger than 75 years of age with low education is < 83; with moderate or high education < 84; for individuals of 75 years of age or older with low education < 65; with moderate or high education < 78 [14, 16]. The cut-off for aMCI for individuals with low education is < 26; with moderate or high education < 28 [12]. As the formal diagnosis of dementia requires the presence of functional impairment in addition to cognitive impairment, we also used Lawton and Brody’s scale on instrumental activities of daily living (iADL) to estimate the presence of dementia [17]. Finally, we used the delirium observation scale to rule out delirium [18].

### Participant recruitment, sampling strategy and data collection

All 647 patients labeled ‘possible cognitive impairment or dementia’ (positive index test) were invited for study participation. From the group labeled ‘no signs of cognitive impairment’ (negative index test) (*n* = 6582), a random, age and gender matched sample of 442 patients was invited to participate. Patients were included between October 2011 and May 2012. Due to a delay in the assessment of our study protocol by the medical ethics committee, the mean time between index and reference test was 8.9 months (SD 4.6 months).

### Outcomes

The accuracy of FPs awareness of cognitive impairment was expressed as PPV and NPV. Since we were interested in cases where FPs were unaware of it, while cognitive impairment was present, we decided to present the 1-NPV instead of the NPV in the results section. The primary comparison was the FPs’ label versus the reference category ‘dementia or aMCI’. The secondary comparison was the the FPs’ label versus the reference category ‘dementia’ alone.

### Determinants of agreement between the cognitive classification by FPs and the reference test

We also assessed whether the following patient characteristics were associated with con- and discordance between FPs’ evaluation of cognition and results of the reference test: gender, age, contact frequency, time registered with FP, living alone or not and level of ADL- and instrumental-ADL dependency.

### Non-response analysis

To assess whether selective non-response occurred, a non-response analysis was performed in a sample of 210 individuals in four Primary Care Practices (PCPs) providing sufficient numbers of both respondents and non-respondents to allow statistical comparison. These PCPs were selected to reflect both ends of the classification spectrum (positive index test rates ranging from 6.0 to 11.6 %).

To test our hypothesis that there may be more individuals with cognitive impairment among non-respondents, anonymous data on non-respondents were gathered. The results of all cognitive tests performed by medical staff as part of standard care were used. To allow comparison, FPs participating in the non-response analysis agreed to use the CAMCOG instead of the MMSE to test the cognitive status among non-respondents, during the time of the non-response analysis. FPs or their staff obtained informed consent to use the anonymous results of the single cognitive tests for study purposes.

### Statistical analysis

Logistic regression was used to assess which patient characteristics other than age and the CAMCOG score were associated with FPs’ judgment on the presence or absence of cognitive impairment. We adjusted for age and for the raw CAMCOG score.

The PPV and 1-NPV were defined as the proportion of patients with FPs’ label ‘possible cognitive impairment or dementia’ that were cognitively impaired according to the reference test and the proportion of patients with FPs’ label ‘no signs of cognitive impairment’ that were not cognitively impaired according to the reference test, respectively [14]. As the sample selected to receive the reference test from the latter group was matched to the group labeled ‘possible cognitive impairment or dementia’ on age and gender, we used sampling weights to estimate the NPV. Sampling weights were taken as the inverse of the proportion of patients within the stratum for which reference test results were obtained. The same strategy was used to correct for non-response in the group labeled as ‘possible cognitive impairment or dementia’. Stata 12 was used to obtain confidence intervals for the weighed diagnostic accuracy measures. Although clustering effects on the FP level were assumed likely, confidence intervals presented were not corrected for clustering due to the small cluster size and binary nature of the outcome.

Logistic regression was used to assess which patient characteristics were associated with con- and discordance between FPs’ evaluation of cognition and results of the reference test.

To explore potential selection bias, we compared age, gender and cogntive status between respondents and non-respondents in four primary care practices using chi-square and t-tests. The odds ratio of dementia *or* aMCI according to the CAMCOG in respondents versus non-respondents was calculated.

To assess whether the time that elapsed between index and reference had any substantial effect on our outcomes, we used follow-up data from the RCT of which this study was part and compared the number of dementia and aMCI cases based on the CAMCOG scores at baseline (this study) and 1-year follow-up (the RCT). In addition, we calculated the OR of a concordant versus a discordant cognitive classification as a function of the time between index and reference test. SPSS Statistics® 20 and Stata® 12 were used for the analysis.

### Ethics committee approval

Ethical approval for the study was obtained from the medical ethics committee of the VU University Medical Center Amsterdam, The Netherlands (reference number 2010/297). The study protocol is in accordance with the principles of the current version of the declaration of Helsinki. Written informed consent was obtained from all study participants.

The STARD criteria for the reporting of studies of diagnostic accuracy were used to write this paper [19].

## Results

### Cognitive classification by FPs

Of all 7.865 elderly in the practices, 83.7 % (*n* = 6582) were labelled ‘no signs of cognitive impairment’ (negative index test) by FPs; 8.2 % (*n* = 647) were labeled ‘possible cognitive impairment or dementia’ (positive index test), 3.6 % (*n* = 284) ‘probable dementia’ and 4.5 % (*n* = 352) ‘unknown or no recent contact’.

### Cognitive classification at practice level

There was substantial variation between practices in the distribution of index test outcomes. FPs classified between 32.3 and 94.7 % of older persons as ‘no signs of cognitive impairment’ and between 2.0 and 21.1 % as ‘possible cognitive impairment or dementia’. However, two younger recently settled FPs appeared outliers with much higher rates of the label ‘unaware or no recent contact’ than other FPs and also relatively high rates of ‘possible cognitive impairment or dementia’. Without these two, the lowest percentage labeled ‘no signs of cognitive impairment’ was 77.1 % and the highest percentage ‘possible cognitive impairment or dementia’ was 14.0 %. Classifications per practice are shown in Additional file 1: Appendix 1, with PCP 12 and 16 being the outliers.

### Family Physician and Primary Care Practice characteristics

Half of the participating FPs (*n* = 29) was female, the mean age of FPs was 49.5 (SD 8.9) years old and they had 16.5 (SD 9.3) years of experience. The average practice counted 3001 (SD 724) patients of whom 16.6 % (SD 6.3 %) were 65 years or older. For comparison, of all Dutch FPs (*n* = 8902) 41 % is female, their mean age is 48.4 (SD 9.0) years and they have 15.1 (SD 10.0) years of experience. The average practice (more than one FP) population size in the Netherlands is 4204 patients with 15.9 % of them aged 65 years or older [20].

### Determinants of cognitive classification by the FPs

As expected, the mean age of individuals with the label ‘possible cognitive impairment or dementia’ (*n* = 6582; mean age 80 (SD 7.3)) was higher than that of individuals with ‘no signs of cognitive impairment’ (*n* = 647; mean age 73.7 (SD 6.5), *p* < 0.001). The percentage of females was 58.0 and 54.7 %, respectively (*p* = 0.11). Patients with more comorbidities (OR 7.46, 95 %-CI 4.00 – 14.08) and a higher level of iADL dependency (OR 1.61 95 %-CI 1.03 – 1.31) had a higher chance of having the label ‘possible cognitive impairment or dementia’ instead of ‘no signs of cognitive impairment’, irrespective of their age and CAMCOG score.

### Response rate

**Fig. 1:**
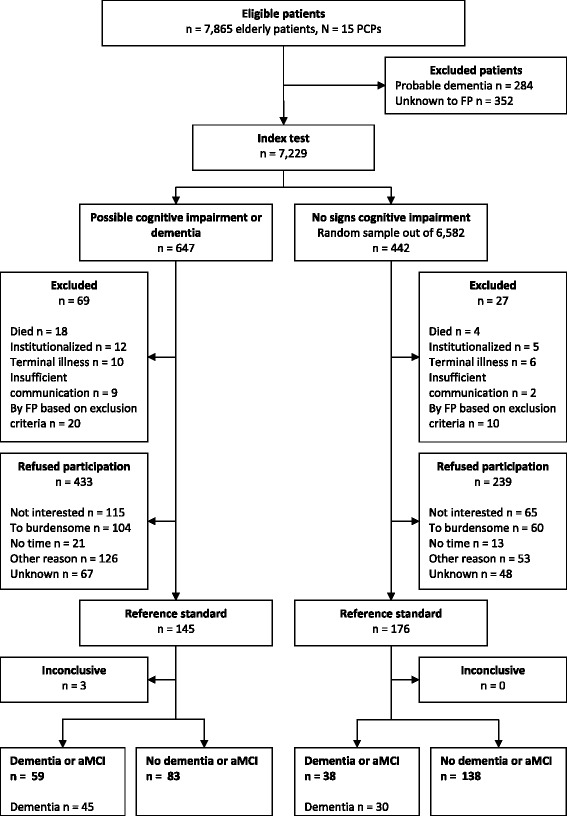
Flow chart of the inclusion process

A complete reference standard was obtained from 142 (22 %) of all patients labeled ‘possible cognitive impairment or dementia’ and from 176 (40 %) of randomly sampled age and gender matched patients with ‘no signs of cognitive impairment’. Figure 1 provides a flow chart of the inclusion process.

### Prior chance of cognitive impairment in all participating elderly

The prior chance of a CAMCOG score below the age and education specific cut-off for dementia and/or a memory section score below the cut-off for aMCI in older persons with a positive and negative index test together was 15.6 %. The prior chance of a CAMCOG score suggestive of dementia was 11.9 %. The prior chance of a CAMCOG score below the cut-off and iADL dependency was 7.6 %.

### PPV of FPs’ label ‘possible cognitive impairment or dementia’ and NPV of FPs’ label ‘no signs of cognitive impairment’

The weighted PPV of the FP label ‘possible cognitive impairment or dementia’ for a CAMCOG score below the age and education specific cut-offs for dementia or aMCI was 47.1 % (95 %-confidence interval: 43.5 – 62.4 %). The 1-NPV of the FP label ‘no signs of cognitive impairment’ for a CAMCOG score suggestive of dementia and aMCI was 12.5 % (95 %-CI 8.2 – 16.8 %). Table 1 provides an overview of the results. FPs’ classification is also compared to the reference category ‘dementia’ alone and presented separately for men and women.

**Table 1:** Overview of correspondence of FP classification with the reference standard

	PPV	PPV women	PPV men	NPV	NPV women	NPV men
CAMCOG suggests:	% (95 % CI)	% (95 % CI)	% (95 % CI)	% (95 % CI)	% (95 % CI)	% (95 % CI)
*Dementia or aMCI*	47.1 (43.5 – 62.4)	55.8 (43.0 – 68.5)	35.3 (24.2 – 46.5)	87.5 (83.2 – 91.8)	87.8 (82.6 – 93.1)	87.1 (79.9 – 94.4)
*Dementia*	34.0 (24.8 – 43.2)	38.1 (27.5 – 48.9)	28.5 (17.9 – 39.0)	90.4 (86.5 – 94.3)	92.1 (87.9 – 96.2)	88.2 (81.1 – 95.4)
*Dementia & iADLd* ^*a*^	25.6 (14.8 – 36.5)	32.5 (22.1 – 32.9)	16.9 (7.3 – 26.4)	93.8 (91.2 – 96.4)	93.0 (89.3 – 96.7)	94.8 (91.5 – 98.0)
Without outliers^b^						
*Dementia or aMCI*	51.9 (41.9 – 61.8)	60.7 (45.7 – 75.7)	40.3 (27.6 – 53.0)	88.2 (83.7 – 92.6)	87.5 (81.3 – 93.6)	89.0 (82.5 – 95.5)
*Dementia*	37.3 (28.4 – 46.2)	41.2 (28.4 – 54.1)	32.2 (19.9 – 44.4)	91.6 (88.0 – 95.3)	92.9 (88.7 – 97.2)	90.1 (83.7 – 96.4)
*Dementia & iADLd* ^*a*^	26.5 (15.3 – 37.7)	31.8 (20.6 – 43.4)	19.8 (8.4 – 30.7)	94.4 (91.9 – 96.9)	94.2 (90.8 – 97.6)	94.6 (91.2 – 98.0)

^a^ Dependency on at least one instrumentalADL item of the Lawton and Brody scale [17]

^b^ Analysis without the cognitive classification of two FPs who were unaware of the cognitive status in a relatively high proportion of elderly (PCP 12 and 16 in the appendix)

### PPV and NPV without outliers

When the two FP outliers were excluded, the PPV of the label ‘possible cognitive impairment or dementia’ for a CAMCOG score suggestive of dementia or aMCI became 51.9 % (95 %-CI: 41.9 – 61.8 %). The 1-NPV of the label ‘no signs of cognitive impairment’ for a CAMCOG score suggestive of dementia and aMCI became 11.8 % (95 %-CI: 7.4 – 16.3 %).

### Determinants of agreement between the cognitive classification by FPs and the reference test

We explored whether gender, age, contact frequency, time registered with FP, living alone or together with others and level of (instrumental) ADL dependency predicted concordance or discordance between FPs’ classification of the cognitive status and classification based on the CAMCOG results.

The chance of discordance between FPs’ label ‘no signs of cognitive impairment’ and the CAMCOG classification increased with age; the OR of a discordant versus a concordant classification was 1.15 (95 %-CI 1.09 – 1.23) per year older.

The chance of concordance between FPs’ label ‘possible cognitive impairment or dementia’ was higher if persons were more dependent of others in their ADL and instrumental-ADL (i-ADL); the OR of concordant versus a discordant classification was 2.24 (95 %-CI 1.16 – 4.35) for persons who were versus persons who were not ADL dependent and 1.69 (95 %-CI 1.26 – 2.27) for persons who were versus were not i-ADL dependent. None of the other characteristics was significantly associated with con- or discordance of the classification.

### Exploration of selective non-response

Respondents were younger and more often male than non-respondents, both in the group where FPs suspected cognitive impairment and in the group where they had not noticed signs of it. The mean ages of respondents and non-respondents with suspected cognitive impairment were 78.1 and 80.7 years, respectively (*p* < 0.01). The mean ages of respondents and non-respondents in whom FPs had not noticed signs of cognitive impairment were 78.6 and 80.2 years, respectively (*p* = 0.02). The proportions of females among respondents and non-respondents with suspected cognitive impairment were 42.1 and 63.1 %, respectively (*p* < 0.01) and among individuals with no signs of cognitive impairment 53.0 and 63.2 %, respectively (*p* = 0.03).

In the four PCPs included in the non-response analysis, there were 53 (25 %) respondents and 157 (75 %) non-respondents. We compared the rate of CAMCOG scores below the cut-off for dementia between respondents and non-respondents. A complete CAMCOG was obtained from 52 respondents (98 %) and 46 non-respondents (29 %). The age and gender corrected OR was 0.97 (95 %-CI 0.51 – 1.87) for a CAMCOG suggestive of dementia in respondents compared to non-respondents.

### Exploration of the effect of time between index and reference test

The odds of a concordant versus a discordant classification were similar among individuals with a short and a longer time between index and reference test (OR 1.05 per month, 95 %-CI 0.96 – 1.13). The development of the CAMCOG outcome of 105 individuals from whom a CAMCOG was obtained at baseline and at 1 year follow-up (data from the RCT of which this study was part) is provided in Additional file 2: Appendix 2. Although there were quite some shifts from normal ageing to aMCI or dementia and vice versa at the individual level, the total number of respondents with aMCI or dementia was comparable at baseline and 1-year follow-up. The McNemar test, applied to compare the percentage with ‘aMCI or dementia’ at baseline and 1-year follow-up (*p* = 1.00) and the percentage with ‘dementia’ at baseline and 1-year follow-up (*p* = 0.82) showed non-significant differences.

## Discussion

### Summary of main findings

FPs labeled 3.6 % of older persons with ‘probable dementia’, 8.2 % with ‘possible cognitive impairment or dementia’, 83.7 % ‘no signs of cognitive impairment’. They could not label the remaining 4.5 % of elderly. Persons labelled ‘possible cognitive impairment or dementia’ had more comorbidities and were more often iADL dependent than persons labelled ‘no signs of cognitive impairment’, irrespective of their age and CAMCOG score.

The CAMCOG did not confirm the presence of either dementia or aMCI in half of the persons in whom FPs considered cognitive impairment possibly present. The CAMCOG indicated some form of cognitive impairment in 12.5 % and suggested dementia in 10 % of persons in whom FPs were not aware of any such signs.

If we translate these figures to the average practice in this study with 3000 patients of whom 16.6 % (*n* = 498) are above the age of 65, FPs would suspect cognitive impairment in 41 persons. The CAMCOG would confirm this suspicion in 19, and would not confirm it in 22 persons. FPs would be unaware of cognitive impairment in 52 persons in which the CAMCOG would suggest such impairment.

There was substantial variation in the percentage of persons per cognitive category between FPs. Since the numbers were too small to provide estimates on PPV and NPV at practice level, it was not possible to determine the impact of case-mix differences on PPV and NPV variation between FPs. FPs who provided care for their population for less than three years could not classify the cognitive status of 35 to 40 % of their older patients.

Awareness of cognitive impairment was associated with ADL- and with iADL dependency; unawareness of cognitive impairment was associated with higher age.

### Strengths and limitations

By using a nested design we guaranteed an accurate representation of the source population in our sample and facilitated correction for age and sex [9, 10]. Furthermore, the method used appears to be appropriate to estimate FPs’ actual awareness or unawareness of dementia and aMCI, since not having to discuss their suspicion with the patient or document it in the medical records likely reduced barriers to identifying someone as possibly cognitively impaired.

A limitation of this study is the low response rate. We found that respondents were younger and more often male than non-respondents, although we could not demonstrate selective non-response related to cognitive status. Nevertheless, since age is strongly associated with cognitive impairment, correction for age may have reduced such bias if present. Previous research demonstrated a lower response rate among individuals with more cognitive impairment [21]. If present, such a selection would result in underestimation of the PPV but also of the 1-NPV.

Another potential limitation was the time, on average almost nine months that elapsed between the index and reference test. Nevertheless, this delay did not seem to have impact on the estimate of the FPs’ accuracy in our longitudinal exploration.

The cognitive classification by the FPs had no absolute demarcation between categories and thus leaves room for different interpretations. This may have occurred if FPs doubted between ‘possible’ and ‘probable’ dementia. We cannot rule out that there were some false positives among elderly labeled with ‘probable dementia’.

The CAMCOG was validated in a population-based, purposive sample of older people with a relative overrepresentation of high age and cognitive impairment based on MMSE scores [22]. As a result, the positive predictive value of the CAMCOG for dementia was likely lower in our study population. Results from another population based study support the hypothesis that the CAMCOG may overestimate the prevalence of dementia [23, 24]. Since we used the CAMCOG as a reference standard, the potential effect on our results would be some underestimation of the PPV of FPs’ cognitive labels but an overestimation of the NPV of their labels. In addition, since the CAMCOG was only validated to measure amnestic MCI and not non-amnestic MCI, our results include only the amnestic MCI group. Further, we found substantial shifts in cognitive classification based on the CAMCOG between baseline and 1-year follow-up. This may have several causes: it may be a result of test-retest characteristics of the CAMCOG, the drop in the cut-off scores at the age of 75 (which caused a category shift in three respondents), the included population with predominantly earlier stages of cognitive impairment, and also treatment of specific conditions (e.g. heart failure) which may improve cognitive functioning in between tests may also explain improved CAMCOG scores [22, 23]. However, since the total rate of CAMCOG scores suggestive of dementia and MCI remained stable we presume that these shifts had no substantial effects on our findings.

One should take into account the impact of the prevalence of a condition on the PPV and NPV. If, for example, the prevalence is very high, the PPV will become higher. Thus, our findings are prevalence specific and their external validity is limited to populations with a similar prevalence of cognitive impairment.

As we focused on persons without a firm diagnosis of dementia and did not verify patients labeled with ‘probable dementia’, we could not compute classical diagnostic accuracy measures (e.g. sensitivity and specificity), limiting the comparability of our results [1, 6].

### Comparison with existing literature

A new finding is that the likelihood of missing cognitive impairment was higher in older elderly. Particularly at earlier stages, FPs may incorrectly attribute signs of cognitive decline (e.g. dependency, forgetfulness) to normal ageing. Conversely, FPs may be preoccupied with the multiple chronic conditions of their older patient, and perhaps overlook diffuse cognitive impairments if present. Further, we confirmed the previously described association between awareness confirmed by a reference test and higher ADL dependency, showing that also at earlier stages, dependency is an important clue to cognitive impairment [25].

In contrast to previous findings, living alone was not significantly associated with unawareness of present cognitive impairment in our study [26]. This may reflect that relatives usually wait to ‘help’ FPs become aware until cognitive impairment reaches a more advanced stage. Neither did we find the association between detection and contact frequency that was demonstrated previously [7]. The milder cognitive impairment and thus more subtle symptoms and possibly less variation in contact frequency of the studied group might explain this.

### Interpretation

We chose the combined reference test outcome ‘dementia or aMCI’ for the primary comparison with the index test because the identification of dementia starts with the recognition of cognitive decline. However, the clinically most relevant reference test for any index test would - completely accurately -indicate those patients who would benefit from treatment of the target condition. In the case of dementia this is not straightforward. There is no curative treatment and pharmacological treatment has only temporary and small effects on cognition and sometimes ADL (and not on other outcomes, like global impression) and is often accompanied by side-effects if tolerated at all [27, 28]. Still, there are reasons to establish this diagnosis: most people would like to know about it, support can be offered and care arranged, the diagnosis may influence medical decisions (e.g. the use of medication adherence aids) and it may affect the extent to which patients can be engaged in such decisions [29–32]. To interpret the different reference test outcomes and to link these outcomes to clinical actions, we would suggest that further diagnostic evaluation should be considered in all persons with a CAMCOG score below the cut-off for dementia, even more so when this is accompanied by iADL dependency. When the CAMCOG score suggests only aMCI, the benefit of further diagnostic evaluation is, due to its variable course, not straightforward in the primary care setting [33]. Still, there are indications that persons with MCI are more vulnerable and FPs may consider a more pro-active approach towards these patients necessary [34–37].

That a substantial proportion of persons in whom FPs’ suspected cognitive impairment scored well on the CAMCOG suggests that FPs find it hard to distinguish between earlier stages of cognitive impairment and normal ageing. This finding contributes to our understanding of un-established dementia diagnoses in primary care. Unsureness about whether cognitive deficits are at all present may increase the barrier to raising the issue during regular consultations with the patient or family. In contrast, although a decreased level of functioning helps FPs to become aware of cognitive impairments, the presence of such functional decline may also be erroneously interpreted as a sign of cognitive deterioration.

That a substantial proportion of persons in whom FPs’ had not noticed signs of cognitive impairment had a CAMCOG score suggestive of aMCI or dementia suggests that complete unawareness of cognitive impairment is a major explanatory factor for missed diagnoses of dementia in primary care. However, this should be seen in context: awareness is not only a result of how capable FPs are of recognizing cognitive impairment, but also of whether or not patients and their relatives bring it to bear (e.g. consultation frequency, discussing versus masking memory impairment) and of how the healthcare system is organized (e.g. time available per patient contact).

Still, FPs’ awareness of cognitive impairment is likely influenced by their own knowledge about symptoms and signs of dementia and by their attitude regarding the value of such awareness. This attitude shows quite some variation: some FPs value establishment of a diagnosis as a very important part of their function towards the patient and family, whereas others feel that provision of good care does not require a diagnosis, or that a diagnosis may even be harmful to the patient [25, 38–40].

### Implications for practice and future research

A vast body of evidence shows that, although patients’ receptiveness to discuss this topic will vary, the large majority will appreciate an open attitude to disclose suspicions of dementia [26]. Relatives, although not unequivocally in favour of disclosure towards the patient, do usually value clarity about the presence of dementia as well [41]. This suggests that FPs may be less hesitant to discuss suspected cognitive impairment.

At present, most guidelines on the diagnosis and management of dementia do not provide guidance for practitioners on how to diagnose, inform about and manage cognitive problems other than dementia, like MCI [42, 43]. We assume that if such guidance would be available, this would decrease barriers for FPs to discuss suspicions of cognitive impairment.

Like with other missed diagnoses, not considering the diagnosis at all may be an important cause of FPs’ limited awareness of cognitive impairment. In this respect, it would be interesting to explore how FPs awareness would develop if they would be primed to the possibility of cognitive impairment, for example by performing the classification procedure every year. As a results FPs may also become more alert to other signs of dementia like mistakes with repeat medication, gait disturbances, weight loss or apathy [44–46].

Awareness may also be improved by measures not directly involving the FP. Other PCP co-workers may be stimulated to communicate about signs of cognitive impairment. Public campaigns aimed to reduce fear and stigma and to inform about available services may help patients or their relatives seek professional help sooner [47–49].

## Conclusion

Evaluation of FPs’ classification of the global cognitive function of elderly without a firm diagnosis of dementia showed both over- and unawareness of the presence of cognitive impairment. FPs were more often unaware of cognitive impairment in the oldest old. In contrast, a higher level of dependency helped them to recognize it. Our findings suggest that unawareness of cognitive impairment may be the main cause of un-established dementia diagnoses in primary care.

## Additional files

Additional file 1: Appendix 1. Graph of cognitive classification per practice. Legend: Primary Care Practice 12 and 16 were the outliers. (DOC 50 kb) Additional file 2: Appendix 2. Number of respondents per CAMCOG outcome category at baseline and 1-year follow-up. (DOC 47 kb)

## Abbreviations

ADL: Activities of Daily Living
aMCI: Amnestic Mild Cognitive Impairment
CAMCOG: Cambridge Cognitive Examination
EMR: Electronic Medical Records
FP: Family Physician
MMSE: Mini Mental State Examination
NPV: Negative Predictive Value
OR: Odds Ratio
PCP: Primary Care Practice
PPV: Positive Predictive Value
RCT: Randomized Controlled Trial
STARD: Standards for the Reporting of Diagnostic accuracy studies
