# Supplementary figures and images for: Dutch family physicians’ awareness of cognitive impairment among the elderly
Source: BMC Geriatr. 2015 Aug 27;15:105. doi: 10.1186/s12877-015-0105-1 (PMC4549900; doi:10.1186/s12877-015-0105-1)

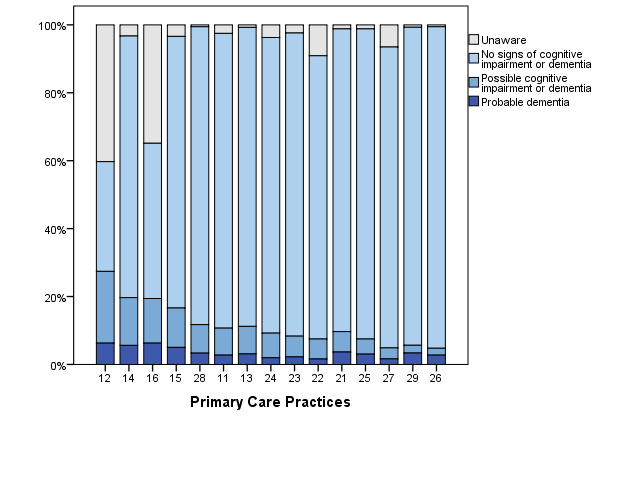

Supplement: Additional file 1: Appendix 1. — Graph of cognitive classification per practice. Legend: Primary Care Practice 12 and 16 were the outliers. (DOC 50 kb) [file 12877_2015_105_MOESM1_ESM.doc]
