# Supplementary material for: Dutch family physicians’ awareness of cognitive impairment among the elderly
Source: BMC Geriatr. 2015 Aug 27;15:105. doi: 10.1186/s12877-015-0105-1 (PMC4549900; doi:10.1186/s12877-015-0105-1)
Supplement: Additional file 2: Appendix 2. — Number of respondents per CAMCOG outcome category at baseline and 1-year follow-up. (DOC 47 kb) [file 12877_2015_105_MOESM2_ESM.doc]

|  |  |  | **1-year follow-up** |  |  |
| --- | --- | --- | --- | --- | --- |
|  |  | normal ageing (n) | aMCI (n) | dementia (n) | total |
|  | normal ageing (n) | 54 | 6 | 6 | 66 |
| **Baseline** | aMCI (n) | 1 | 7 | 2 | 10 |
|  | dementia (n) | 10 | 0 | 19 | 29 |
|  | total | 65 | 13 | 27 | 105 |
